# High-resolution mapping based on an Unmanned Aerial Vehicle (UAV) to capture paleoseismic offsets along the Altyn-Tagh fault, China

**DOI:** 10.1038/s41598-017-08119-2

**Published:** 2017-08-15

**Authors:** Mingxing Gao, Xiwei Xu, Yann Klinger, Jerome van der Woerd, Paul Tapponnier

**Affiliations:** 1 Earth Observatory of Singapore, Nanyang Technological University, Singapore, 639798 Singapore; 2 Key Laboratory of Active Tectonics and Volcano, China Earthquake Administration, Beijing, 10029 China; 3 Institut de Physique du Globe de Paris UMR7154, 1, rue Jussieu, 75238 Paris, Cedex 05 France; 4 IPGS-EOST UMR7516 CNRS/Université de Strasbourg, 5, rue Rene Descartes, F-67084 Strasbourg, Cedex France

## Abstract

The recent dramatic increase in millimeter- to centimeter- resolution topographic datasets obtained via multi-view photogrammetry raises the possibility of mapping detailed offset geomorphology and constraining the spatial characteristics of active faults. Here, for the first time, we applied this new method to acquire high-resolution imagery and generate topographic data along the Altyn Tagh fault, which is located in a remote high elevation area and shows preserved ancient earthquake surface ruptures. A digital elevation model (DEM) with a resolution of 0.065 m and an orthophoto with a resolution of 0.016 m were generated from these images. We identified piercing markers and reconstructed offsets based on both the orthoimage and the topography. The high-resolution UAV data were used to accurately measure the recent seismic offset. We obtained the recent offset of 7 ± 1 m. Combined with the high resolution satellite image, we measured cumulative offsets of 15 ± 2 m, 20 ± 2 m, 30 ± 2 m, which may be due to multiple paleo-earthquakes. Therefore, UAV mapping can provide fine-scale data for the assessment of the seismic hazards.

## Introduction

High-resolution three dimensional (3D) data are essential for constraining the spatial characteristics of active faults as they provide a fundamental dataset for mapping the complexity of faulting and the details of the surficial features offset by the faults. The spatial characteristics of a fault can be addressed using datasets with different resolutions^1, 2^. Sub-meter-resolution satellite images (QuickBird, GeoEye, Pleiades, etc.) and airborne Lidar data are commonly used to map earthquake surface ruptures over hundreds of kilometers. However, the spatial and vertical accuracies of these datasets are not comparable to more precise measurement methods, such as surveys using total station, real-time kinematic Global Positioning System (GPS) or terrestrial Lidar data^3^. Still, the acquisition of such data requires long survey sessions and significant investments of time in data processing, and such acquisitions are not always possible due to logistics.

With the development of remote sensing devices and new mapping techniques, the Unmanned Aerial Vehicles (UAV) have been used to enhance the mapping efficiency and to obtain images and 3D data at an unprecedented resolution^4, 5^. UAV mapping, like other photogrammetry-based techniques (i.e., terrestrial digital photogrammetry), captures 3D information of features from two or more photographs of the same object, taken from different angles^6–8^. High-quality photos can be quickly and easily collected in the field by attaching modern digital cameras to a small flying platform. The platform is generally inexpensive and can be either a fixed-wing type or a multi-rotor UAV. Multiple investigations have used UAVs to map folds and fractures in low elevation regions^9–12^. However, using UAV techniques to map faults in high elevation areas is rare, mainly due to poor accessibility to the field sites and the difficulties associated with proper logistical support. In addition, operating UAVs at high elevated sites can be challenging due to issues such as lower air pressure^13–15^ and frequent signal disturbances, as the level of ionization increases with elevation.

**Figure 1:**
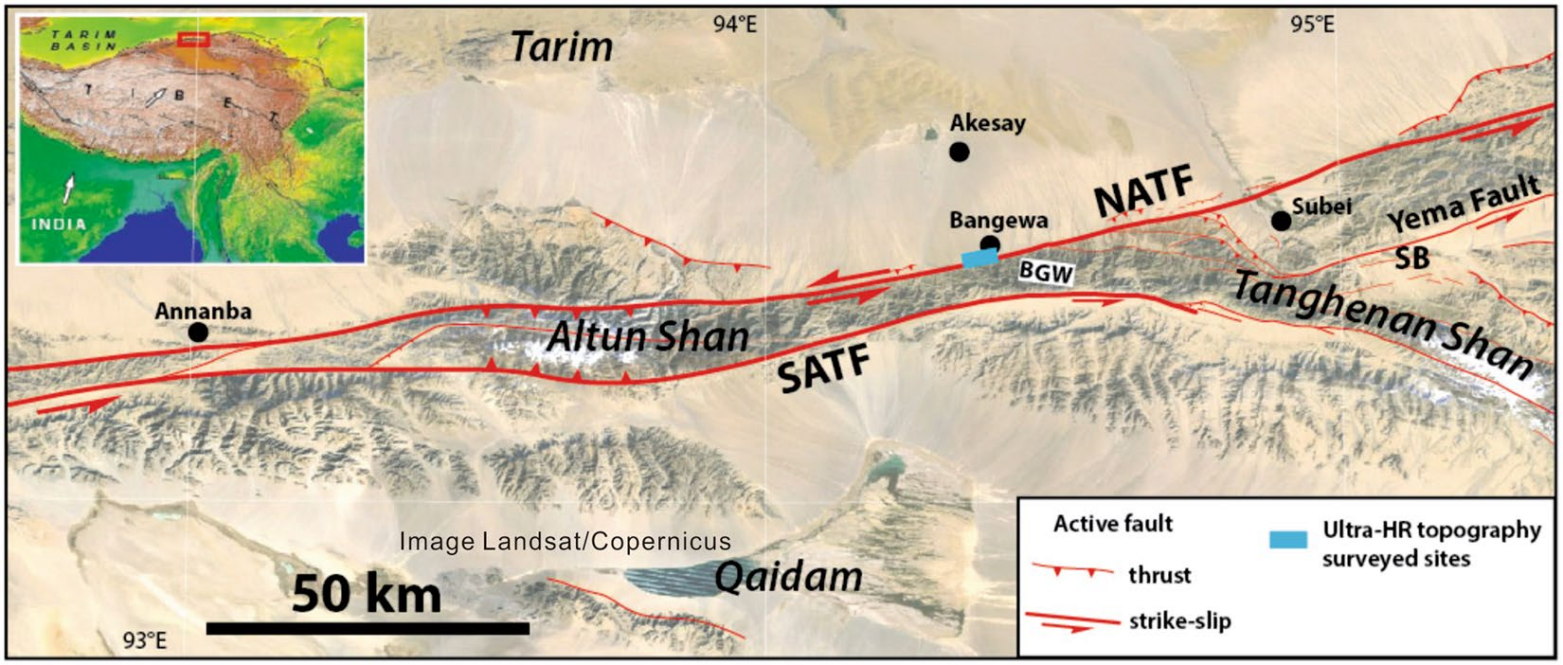
Overview of the northeast section of the Altyn Tagh fault (see red rectangle in the inset for location). NATF: North Altyn-Tagh fault, SATF: South Altyn-Tagh fault. The study site is located on the NATF, at the Bangewa site (BGW). Active faults were mapped onto a background image from Google (Image Landsat/Copernicus) based on the Adobe Illustrator mapublisher (http://www.avenza.com/mapublisher). The inset map background is based on a global digital elevation model (GTOPO30 DEM with a horizontal grid spacing of 30 arc seconds, courtesy of the U. S. Geological Survey).

**Figure 2:**
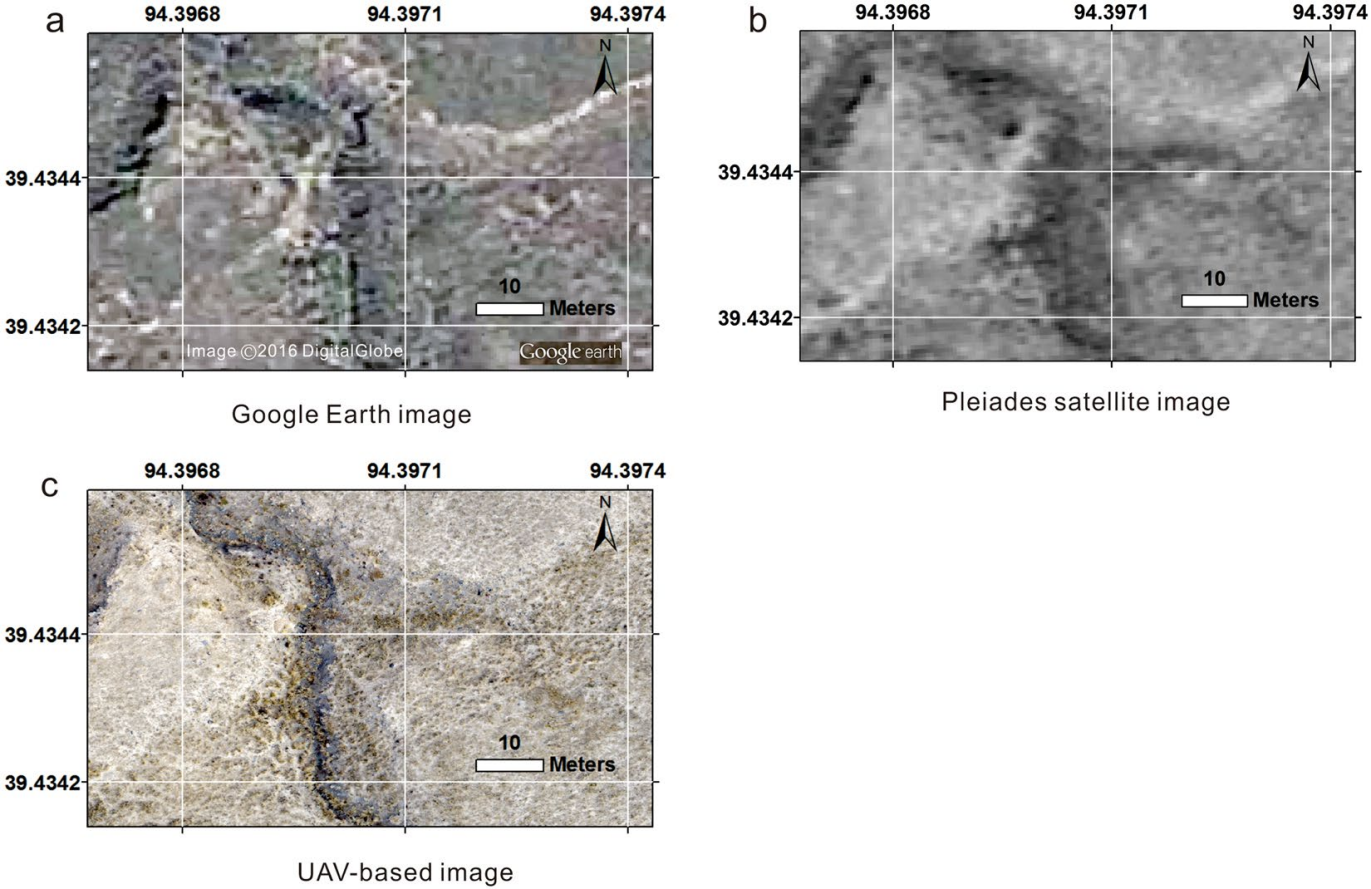
Comparison among (**a**) a Google (DigitalGlobe), and (**b**) a Pleiades satellite image (0.7 m resolution), and (**c**) the UAV image obtained by this study (0.016 m resolution).

**Figure 3:**
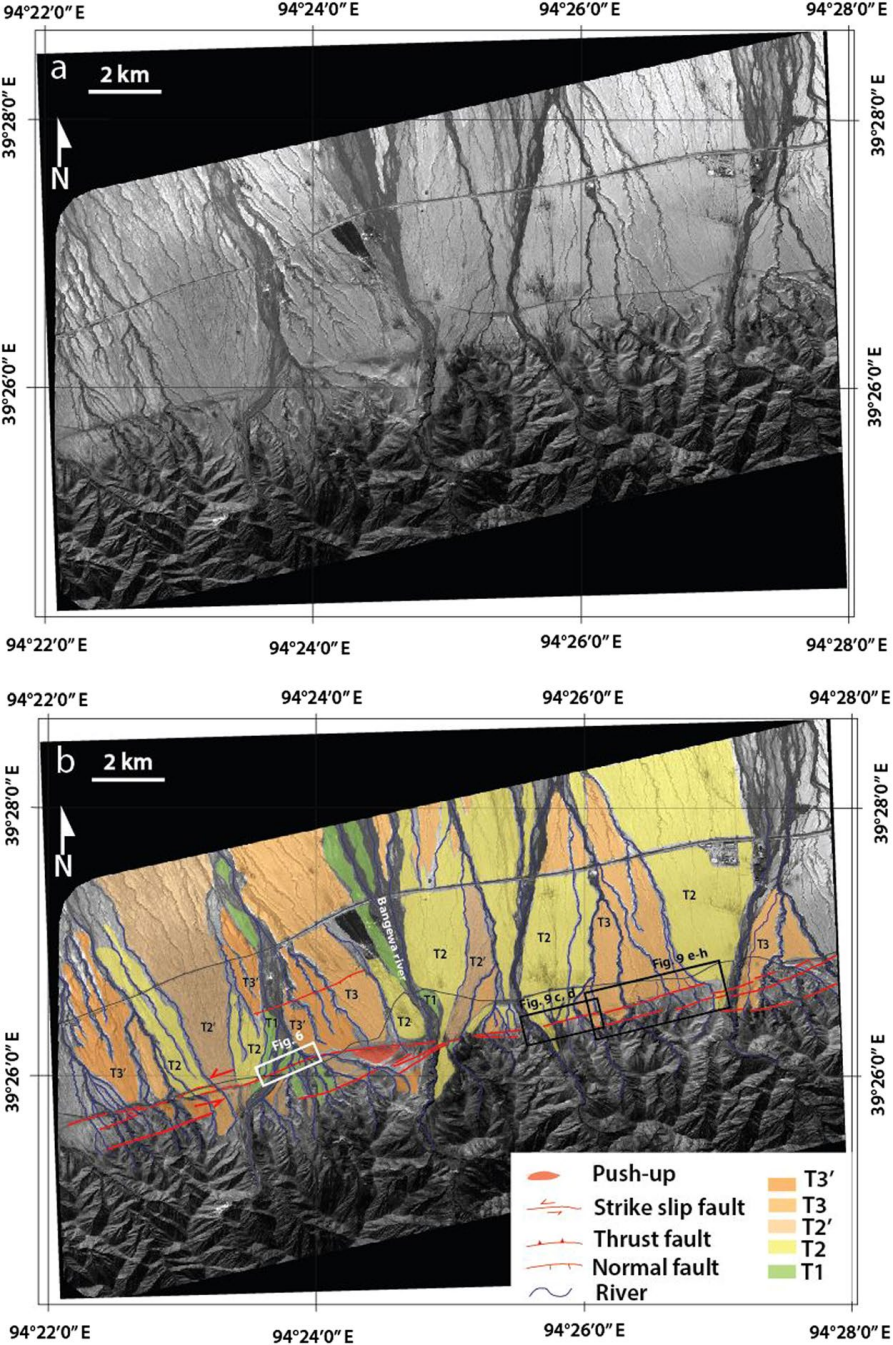
(**a**) Pleiades satellite image (0.7 m resolution) of fans along the ATF near Bangewa. (**b**) Geomorphic interpretation of faulting across the fan apexes and river channels along the fault (revised after Meriaux *et al*.^16^). White boxes indicate the locations of Fig. 6. Black boxes indicate the location of Fig. 9c,d and e–h.

**Figure 4:**
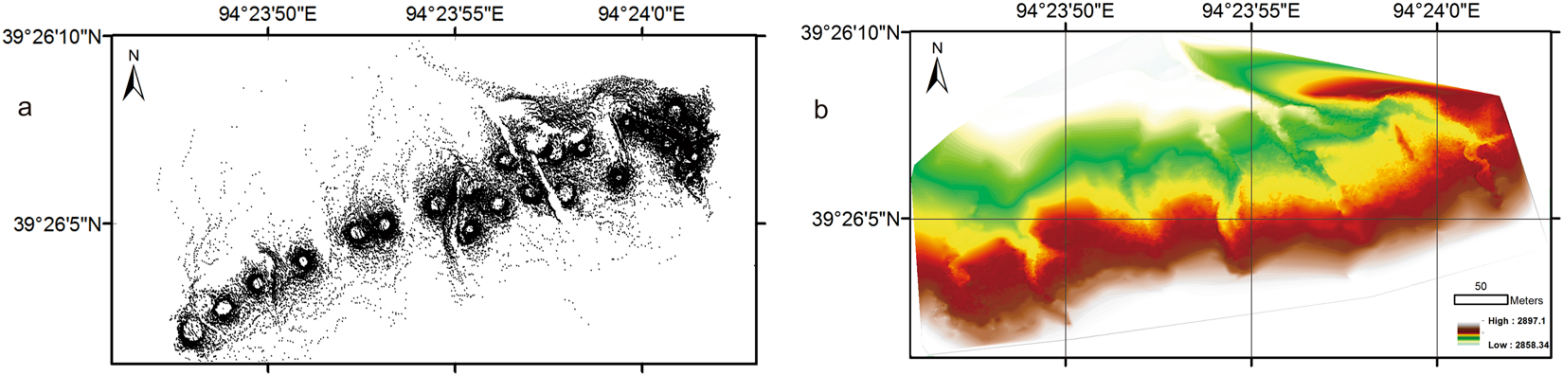
(**a**) Point cloud from Trimble VX spatial station scanning. (**b**) Interpolation of the digital elevation model (0.27 m resolution) based on (**a**).

In this study, we used a multi-rotor UAV that can fly at exceptionally low altitudes with high quality cameras. We aim to use the UAV to capture the evidence of paleo-seismic deformation along an active fault, the Altyn Tagh fault (Fig. 1). This fault is one of the largest strike-slip faults on Earth. We selected the Bangewa segment, where degradation of the landscape is minimal due to the dry climatic and where earthquake offsets are consequently well preserved. The well-preserved co-seismic and cumulative offsets, which characterize the spatial displacements along the fault, provide important information for assessing the regional seismic hazards. However, with the currently available satellite images (Fig. 2a,b and c), it is difficult to interpret the offset created by the most recent earthquake (MRE) or small cumulative offsets. Therefore, targeting this problem, our investigation aims to obtain high resolution data to provide accurate measurements of these recent offsets. Additionally, we validated the cumulative offsets based on SPOT satellite images obtained by Mériaux *et al*.^16^. In this study, we first introduce the UAV methodology used to obtain the images and topographic data. Then, we reconstruct the offsets based on the new data. Finally, we evaluate the results by comparing them with satellite images (Figs 2 and 3) and terrestrial laser scanning (TSL) results (Fig. 4). The methods used in this study can be applied to any active fault, and the results of the study reveal the best methods for efficiently mapping the details of fault ruptures in high resolution.

The Bangewa site (BGW) is located along the North Altyn Tagh fault (NATF) (Fig. 1). This is a fault where large earthquakes have not been recorded in the historical earthquake catalog or by modern instruments. This region lies in the rain-shadow of the Himalayan range, and its inhabitants are predominantly nomadic. Here, the effects of climatic or anthropogenic degradation on the landscape are minimal. As a result, it represents an extraordinary “museum” of extremely well preserved historic and ancient strike-slip ruptures^17^. The fault system comprises two principal parallel strands bounding the Altun Shan, which represents a large push-up structure. The South Altyn Tagh fault (SATF) follows the western rim of the Qaidam basin. The North Altyn Tagh fault (NATF) cuts through the Altun Shan with a N84°E strike (Fig. 1). Systematic sinistral offsets of alluvial fans, fluvial terraces, risers and channels are observed all along the fault, whose principal trace is remarkably sharp and linear, indicating sustained active tectonic movement (Fig. 3). We conducted this UAV-based investigation to further constrain the spatial characteristics of the ATF, to validate the offset obtained by the previous study, i.e., approximately 20 m^16^, and to measure the recent small seismic offset.

## Methodology

To obtain high resolution topographic data at the Bangewa site, we followed the typical procedures^5^ of using a UAV. Here, we first introduce the UAV platform and the workflow used to acquire high resolution topographic data. Then, based on the acquired data, we accurately reconstruct the offsets created by the recent earthquakes.

### Platform and sensor

The self-assembled UAV platform used in this study is equipped with a Sony NEX 5 T digital camera with a Sony Alpha Wide-Angle E-Mount lens, which has a 16 mm focal length and a 16 megapixel resolution. The whole platform weighs approximately 3 kg, including the 3300 mAh battery. The total cost of the equipment is less than $4,000 USD. A single flight conducted at approximately 60 m above ground level can produce approximately 200–250 images using a standard operating configuration. Detailed specifications are listed in Table 1. The Hexa-copter has an onboard navigation system based on a navigation-grade GPS receiver (NAZA-M2). Imaging is triggered by a camera auto-trigger at a rate of approximately 1 photo/s, which provides ample image overlap.

**Table 1:** Key specifications of the self-assembled Unmanned Aerial Vehicle (UAV) system.

Item	Specification
Frame	Carboncore, UK
Type	Hexacopter Y6 950 CO-AXIAL
Engine Power	6 Tahmazo Pro. C Max-30A speedcontroller
Domain	DJI NAZA-M V2 with GPS
Dimension and weight	120 cm, 2.7 kg
Flight mode	Wireless control
Endurance	Standard 4.5 min (±30 seconds safety)
Flexible camera configurations	Digital gimbal, Sony NEX T (mounted with Sony Alpha Wide-Angle E-mount lens), res. 4912 × 3624, pixel size: 4.89089 × 4.89089 um
Ground Control	Futaba 8J, FHSS RADIO W/R2008SB
Propeller size	14 inch
Battery	Baopai 6S 3300 mAh 22.2 V

### Workflow for high-precision data collection

**Figure 5:**
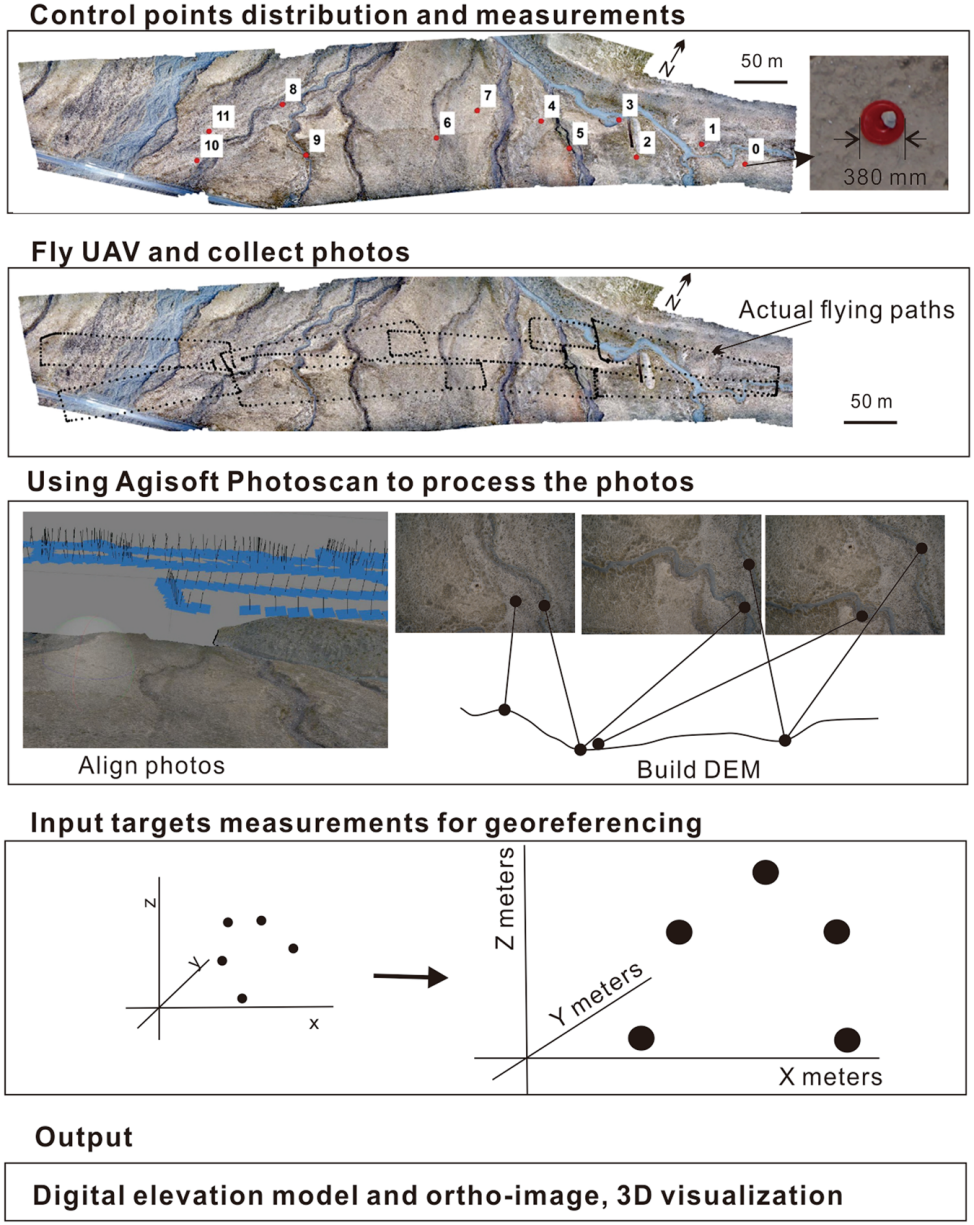
Schematic illustration of the workflow of photo-based mapping procedures using a UAV and the Agisoft Photoscan software. Control points (red dots) were measured using a Trimble handheld GPS with a high-sensitivity GPS receiver.

Multi-view photogrammetry works on the basis that two or more overlapping photographs can be used to 1) calculate the unique three-dimensional (3D) location of a set of homologous image points in both photographs, which in turn can be used to compute the position and orientation of the camera, and 2) obtain a mosaic orthoimage. Photos are typically collected with at least 50–60% overlap under near-parallel viewing conditions^18, 19^. In this study, we used the Structure from Motion (SfM) approach, an alternative to the classical digital photogrammetry approach based on the structured acquisition of images^20^. The process starts by considering the scaling and georeferencing requirements for the target surface^2^ (Fig. 5). Although the target surface can be fully reconstructed in 3D without any scale or position information, in order to extract oriented and scaled data, additional control data or direct georeferencing must be used. At least three ground control points (GCPs) are required to perform the scaling or referencing. However, to ensure accuracy in scale and full georeferencing, more GCPs are needed. These GCPs should be distributed widely across the target area^21^. To ensure visibility, 12 bright red plastic boards were used for the GCPs in this study, and the center point of each board was measured. We made two rounds of GPS observations (moving from GCP1 to GCP12 then repeating the process) using the same set of handheld GPS instruments. The measurements were completed within 40 minutes. We calibrated the two rounds of observed data for the final geo-referencing coordinates with differential correction to within 1 m accuracy. The GCPs were collected using a Trimble handheld GPS device which mounted with high-sensitivity GPS receiver. To minimize the horizontal error, each measurement was taken with at least 8 satellites. To quantify the geometric accuracy, the root mean square error (RMSE) was used^22^. The RMSE is the comparison of real world (ground truth) information to estimated (image-derived) measurements. The RMSE values of the GCPs were 0.38 m (X) and 0.26 m (Y) in the horizontal directions and 0.07 m (Z) in the vertical direction. The mean re-projection error was evaluated as approximately 1.1 pixel.

At the study site, wind speeds generally increase over the course of the day, and the UAV flights were therefore performed in the morning to maximize flight stability and image quality. The UAV was launched from a wooden board (1.2 m × 2 m) and was manually flown in a rectangular path (Fig. 5). All of the nine flights performed were successful and yielded imagery usable for both the full coverage of the fault segment and the DEM generation. The images collected in the field were processed with the commercial software, Agisoft Photoscan Professional, following the detailed procedures introduced by Lucieer *et al*.^23^ (Fig. 5). The initial bundle adjustment formed a dense 3D point cloud of the terrain with 37 million points. A more detailed 3D model with 2 million facets was also reconstructed based on multi-view stereopsis. We obtained a high-resolution grid-based DEM with a vertical resolution of 0.065 m, and an orthophoto with a spatial resolution of 0.0016 m. The area of the mapping site is ~700 m long and ~180 m wide. The specific algorithms implemented in Photoscan and the commonly used parameters are described in Verhoeven^24^.

### Reconstruction of the seismic offsets

A seismic offset is the displacement created by an earthquake^12, 17, 25, 26^. The main goal of the reconstruction of seismic offsets is to link the lateral displacements with individual paleoearthquakes. Researchers use imagery to observe and measure the earthquake produced surface offsets, which include the offset caused by the most recent earthquake (MRE) and the cumulative offsets^27, 28^. To document the distribution of offsets along the rupture trace of the fault, we reconstructed them based on high resolution orthoimages combined with the topographic profiles derived from the DEM to assist in the interpretation and measurement of the offset markers, which are mostly terrace risers and river channels. The offset of these features can only have accumulated since the abandonment of the terrace tread, assuming that the feature is contemporaneous with the terrace.

**Figure 6:**
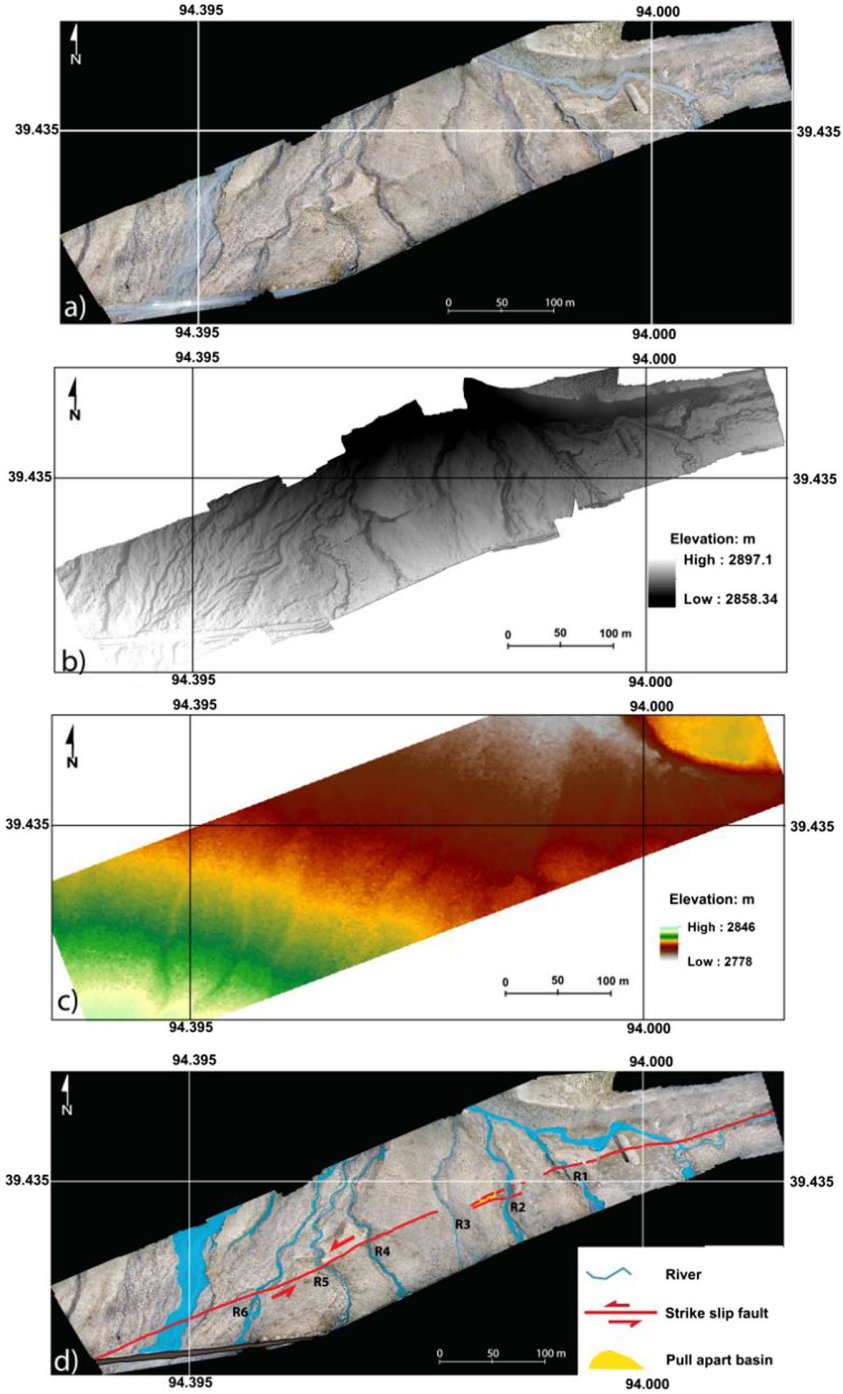
**(a)** Orthoimage (0.016 m resolution) of the study area. (**b**) High resolution bare earth digital elevation model (0.065 m resolution) from the UAV mapping. (**c**) Digital elevation model (0.5 m resolution) calculated from a stero pair of Pleiades satellite images. (**d**) The fault and the six streams (R1–R6) used in the reconstruction of the seismic offsets.

**Figure 7:**
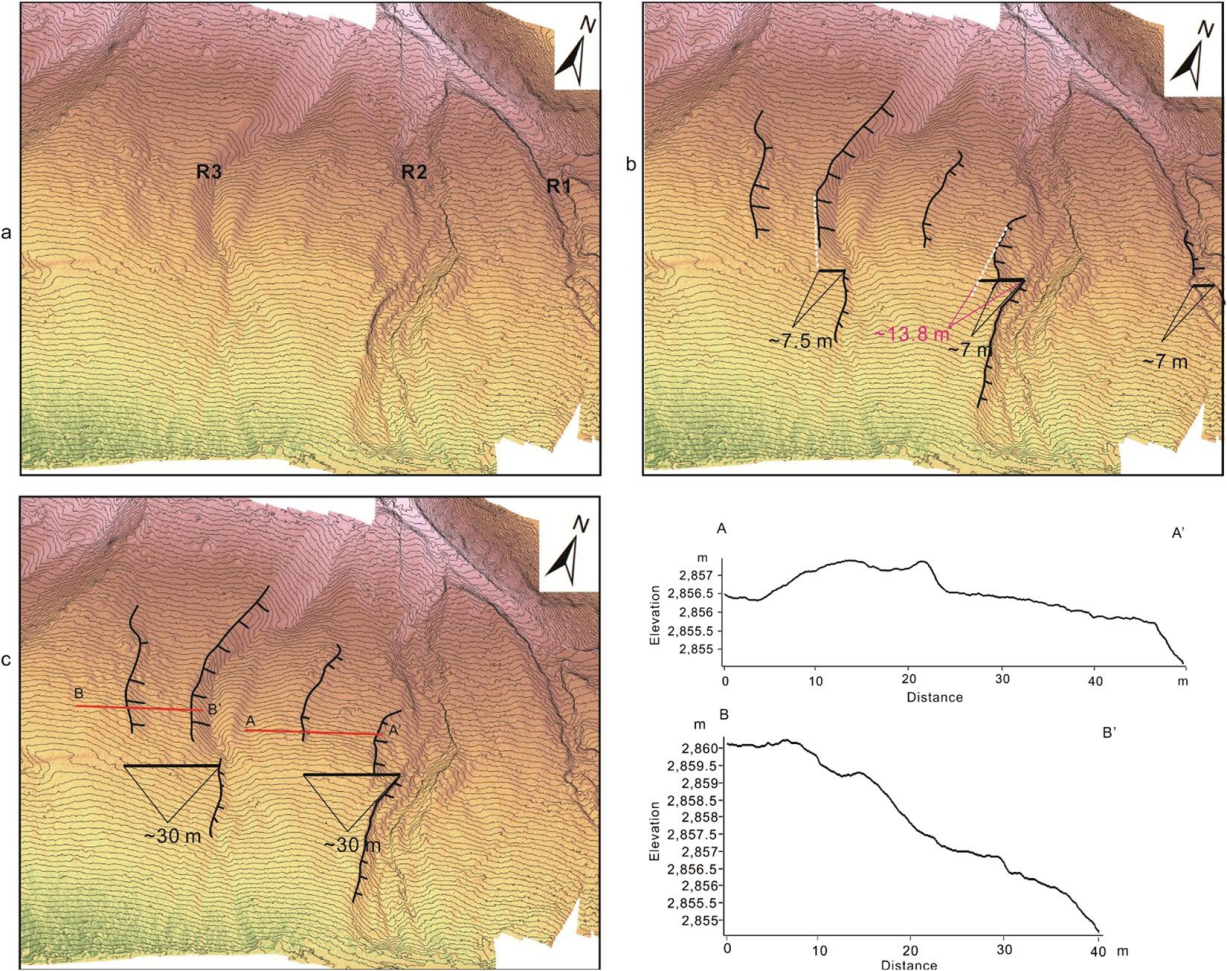
(**a**) Digital elevation model with contour lines (0.25 m interval) for R1, R2 and R3. (**b**) Interpretation of terrace risers and reconstruction of the ~7 m offset. (**c**) Reconstruction of the ~30 m offset.

**Figure 8:**
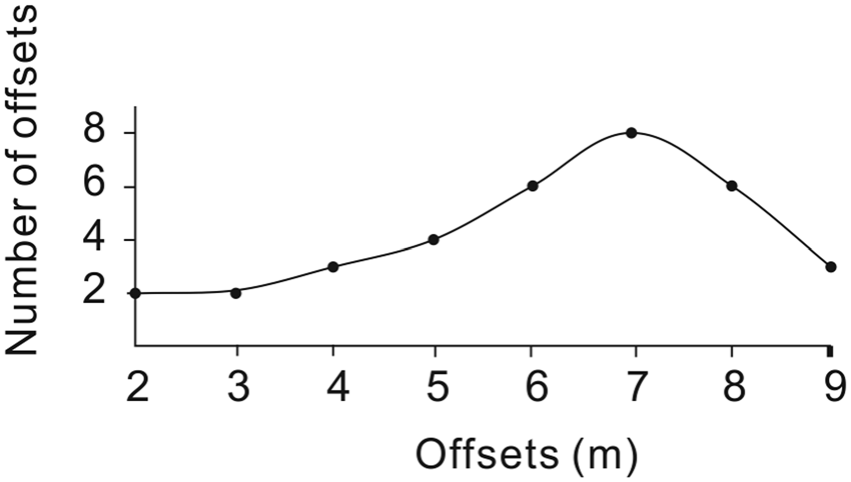
Number of matching markers to reconstruct the offsets from 2–9 m. The maximum markers correspond to an offset of ~7 m. We determined the uncertainty to be ±1 m as we found almost the same number of matching markers for the offsets of 6 m and 8 m.

The fault trace is mapped and the preferred offset values are determined by realigning each marker (terrace riser, gully, and abandoned channel) that has been displaced by fault motion based on both image interpretation and the topographic data (Fig. 6a,b and d). Targeting the clearest offset geomorphic features, we determined the seismic offsets (Fig. 7) by linearly projecting the correlative markers into the fault trace by eye and measuring the range of horizontal offsets^29^. First, we interpreted/identified the geomorphic markers that have been displaced by the motion of the fault. Then, we cut the image along the fault lines and realigned the offset markers. Finally, we measured the length of offset caused by motion on the fault. To evaluate the uncertainty of the recent small offset, we carefully checked the image for offsets from 2 m to 9 m (Fig. 8). To evaluate the uncertainty of the cumulative offset, we referred to the method of Gold *et al*.^29^. We considered two projections for each marker: one based on the trend of the terrace riser/center of the river channel nearest the fault line and a second based on the average trend for the entire length of the terrace riser/center of the river. The error bars for these measurements were determined based on the maximum value between the best fit measurements and the maximum/minimum measurements^26, 29^.

## Results

### Recent small offset obtained from the UAV data

Along the fault at the Bangewa site, geomorphic piercing lines defined by stream channels and terrace risers were identified to measure the offsets. The degree of preservation of offset geomorphic markers was not constant from river channel (R) R1 to R6, and offset markers were not preserved in the most active out-wash. However, by using the high resolution data, especially the contour lines derived from the DEM, we were able to identify micro-scale geomorphic markers, which helped to add important constraints on the small offsets (Fig. 7). Restoring the terrace risers on the left bank of R2, the small offset of ~7.5 m was measured (Fig. 7). At R1, the terrace riser on the left bank is offset by ~7 m (Fig. 7a,b). We carefully checked the entire image for possible offsets of 2 m to 9 m. We found maximum markers for offset of ~7 m (the most clear matching markers are also shown in Fig. 7). We determined that the uncertainty was ±1 m, as we found almost same number of markers for offsets of 6 m and 8 m (Fig. 8).

### Cumulative offset

**Figure 9:**
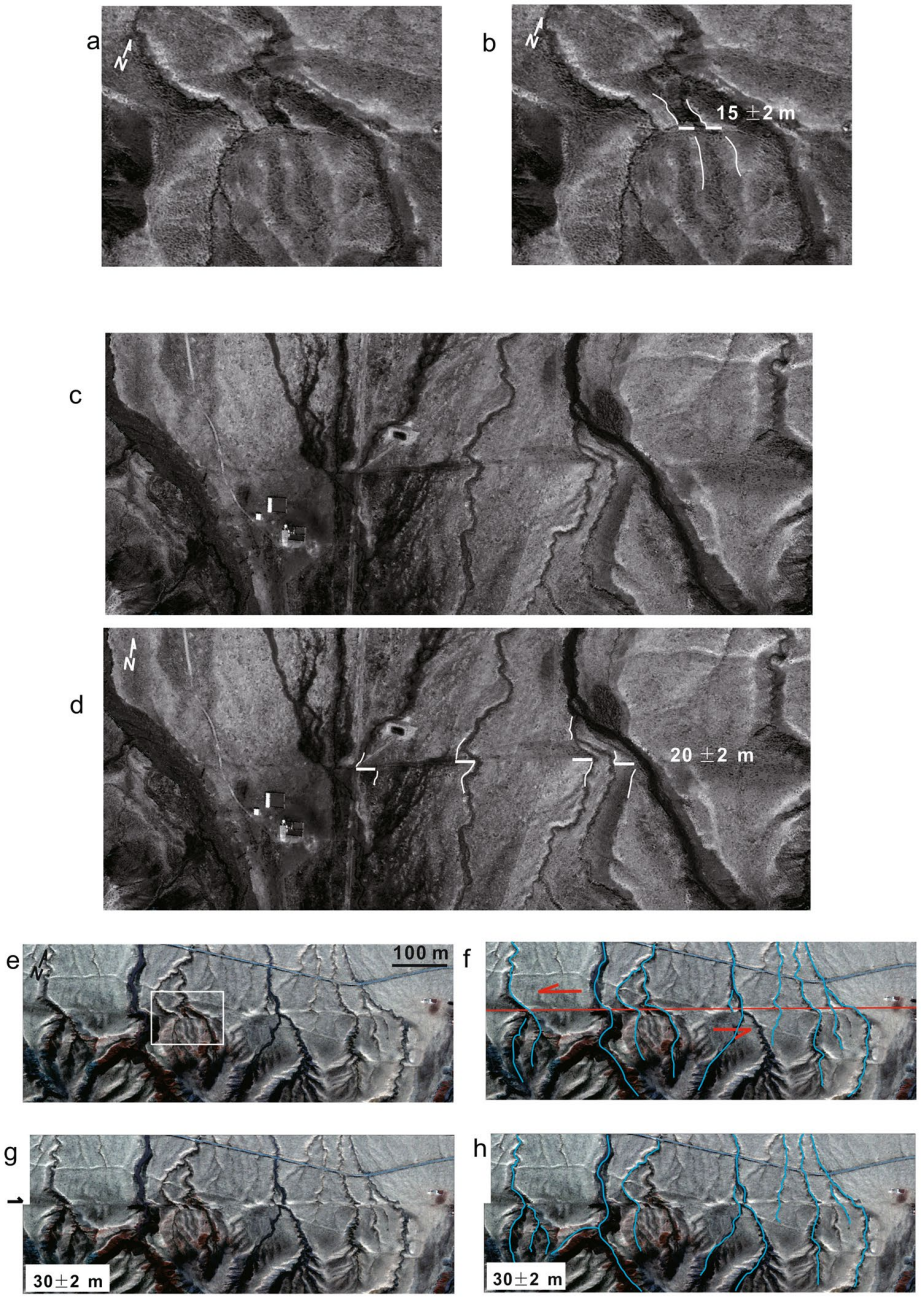
Reconstructions of cumulative offsets based on the Pleiades satellite image. Locations of **a** and **b** were indicated as white box in **e**. See locations of **c**, **d** and **e**–**h** in Fig. 3. Cumulative offset of 15 ± 2 m, 21 ± 2 m and 30 ± 2 m were identified.

We further restore the highest terrace risers of R2 and obtained a cumulative offset of 30 ± 2 m (Fig. 7c). Additionally, we also restored the major active channels and the abandoned channels east of the study site based on the Pleiades satellite image (Fig. 9). Large offsets of 15 ± 2 m, 20 ± 2 m and 30 ± 2 m were obtained. However, the observation of small offsets was difficult due to the relatively low resolution of the satellite image compare to that of the UAV data.

## Discussion

Compared with other remote platforms such as satellites, UAVs can operate at low altitudes^30^. The UAV used in this investigation is capable of flying at low altitudes of approximately 60 m above the ground. Therefore, even on cloudy days or in the early morning when the wind is usually weak and light is not sufficient, UAVs can provide images with unparalleled spatial resolution compared to satellite images (Fig. 2). The high spatial resolution of the data enables researchers to study active tectonics to make more detailed interpretations and measurements of the faulted landscapes^12^.

The resolution and accuracy of the dataset are ideal for identifying micro-scale geomorphic features for offset reconstruction. The uncertainties in the horizontal offset measurements from high resolution satellite imagery restorations are usually greater than 10%^31^. Previous studies using satellite images obtained a smallest cumulative offset value of approximately 20 m^16^. We re-measured this offset and further constrained the uncertainty to be 20 ± 2 m, which is consistent with the previous measurements.

The accuracy of the measurements derived from the imagery is strongly correlated with the acquisition height above the ground^32, 33^. The horizontal RMSE is less than 40 cm, and the vertical RMSE is at the millimeter scale. The errors are small regarding the measurements of the offsets of large earthquakes, which are commonly on the order of a few meters. The main error in the offset is mainly related to how well an offset geomorphic marker is preserved or its shape across the fault trace. The error tends to be larger for eroded markers than for straight markers, such as a linear incised stream or a riser, because the reconstructions of their initial geometry usually involves less ambiguity^17^. However, uncertainties can be reduced by using images in combination with the DEM data to identify the markers, as the relative chronology of the incised features can also be considered using elevation. In this study, we use both imagery and topographic data to reconstruct the offsets; therefore, the accuracies of the UAV-obtained orthoimage and the DEM are sufficiently high for the purpose of seismic offset measurements.

We selected 6 streams along the fault in the Bangewa site to reconstruct the local offsets. The offsets reported here are all from natural terrace risers and fluvial channels, and no man-made features were measured. Uncertainties related to the measurements are mostly due to the degree of preservation of the piercing line across the fault zone. Offsets are not regularly distributed along the fault due to the uneven preservation of the geomorphic markers. All 6 selected streams have been incised by active channels, which are progressively offset by the movement of the fault. Targeting the best-preserved markers and unambiguous offsets ensures that the measurement uncertainties are usually small^17, 26^. As offsets of stream channels can be interpreted in terms of individual paleoearthquakes only if the stream re-establishes its course across the fault between each earthquake^25^, we only use small abandoned streams that are unambiguously related to earthquakes.

The reconstruction uncertainties depend on the marker size and sharpness, the orientation relative to the fault trace and the width of the fault zone. Previous works have suggested that field measurements tend to provide minimum offset values^34–36^. In certain cases, researchers using field observations may mis-interpret the cumulative offset as a single event offset, thereby overestimating the latter^37^. Here, the relative simplicity and freshness of the rupture trace and the high quality of the images make the measurements more reliable. The offset reconstructions for this site were based on a combination of river channels and terrace risers. For reconstructions of successive fluvial risers, criteria associated with the use of either upper or lower terraces should be considered according to the actual geomorphic settings^38^. These criteria should be considered to determine the slip-rates when the ages of the different terraces (both lower and upper terraces) are available. Unfortunately, this study site contains few materials that can be dated with high temporal resolution.

To evaluate the DEM derived from the UAV, a survey using a Trimble VX Spatial Station was performed for the eastern part of the study site (Fig. 4)^39^. The Trimble VX Spatial Station was remotely operated by a TSCII Controller, scanning up to 15 data points persecond. Using infrared direct (ID) reflex technology, this instrument is capable of advanced optical surveying, metric imaging, and 3D scanning, all of which can be accessed in the field through the controller using the real-time video images captured by the station^40^. Since the Trimble VX Spatial Station has an estimated horizontal beam divergence of 4 cm per 100 m and a vertical divergence of 8 cm per 100 m, the scan resolution was typically set to 5 cm horizontal to 2.5 cm vertical, which is more than 10 times lower than the resolution of the UAV-obtained data. Compared to UAV mapping, this mapping technology requires more manpower and more time. In addition, the price of the equipment is also much higher than that of the UAV. The DEM (0.5 resolution) derived from the high resolution images revealed even less detail (Fig. 6c).

Compared with terrestrial Lidar and detailed topographic mapping using total-station instruments, the UAV survey is more efficient, as a minimum of only 1 person is needed to perform the task in the field for a similarly sized mapping area. The price of the terrestrial Lidar is also much higher than that of the UAV. Lidar collects a 3D point location with a single pulse of light and is thus capable of collecting ground surface points wherever the pulse of light is able to penetrate the vegetation, reflect off the ground surface, and return to the instrument. In densely vegetated areas, Lidar may yield higher quality DEMs.

To summarize, it is important to design a survey and choose the mapping tool that suits the resolution needed for the investigation. The methods initially developed to quantify seismic offsets in this study and in similarly little vegetated areas are repeatable and can be applied to the rest of the fault after further testing and improvements. Still, the UAV system is not fully automated and requires the user to make decisions. Future studies may offer an automated UAV approach that minimizes the required level of user attention. Detecting geomorphic markers and measuring seismic offsets could also be processed automatically for large areas^28^.

Based on the restoration of images and 3D topography, we obtained the recent small offset of approximately ~7 m in the UAV data. It is uncertain whether offsets smaller than 7 m have been eroded or not. Although in general, strike-slip faults typically produce less relief than dip-slip faults, they are less likely to destroy the evidence of slip via uplift or erosion, or to disrupt sedimentation, erosion, or other surficial processes that produce stratigraphic and geomorphic markers^25^. However, previous work shown that the offset of the most recent earthquake (MRE) for the south ATF was approximately ~5 m^41^. The slip-rates of the fault were also suggested to be decreasing eastward as the fault keep splaying to the east as thrusts^42^. Therefore, the possibility of that the record of the MRE may not be preserved. Based on the UAV and satellite images, the offset values of approximately 15 m, 20 m and 30 m imply that the offsets of past earthquakes were almost constant along this segment of the fault and might indicate characteristic slip behavior^43^.

There is no large earthquake (M ≥ 7) has been recorded along this segment of the ATF^44, 45^ for a considerable amount of time. The latest paleoseimological investigations on this segment of the fault suggest that the timing of the two MREs occurred at 1200 yr B. P., and 700 yrs B. P., respectively^39, 43^. Nevertheless, the seismic hazard along this segment of the fault is considerable, and further detailed work is needed to date these paleo-events. This work would have implications for the seismic risk posed by a large seismic event for the cities of Dunhuang, Akesay and the Mogao cave, which are approximately 50 km from the fault.

## Conclusions

This study focused on an approximately 1-km-long active fault section in the northern Tibetan Plateau. UAV techniques have been shown to be a powerful tool for mapping active faults at high elevations due to the ability to collect large quantities of high-resolution 3D geospatial data. A significant achievement of this study is that it demonstrated that it is possible to generate accurate DEMs of the fault at high elevations using UAV. The method is fast, and the resolution of the data is high. Along the fault, we measured the most recent seismic offsets resulting from past large earthquakes and reached the following conclusions:

1. Based on the high resolution data, we not only located the fault accurately but also reconstructed the offsets with topography, which enabled us to detect more geomorphic offset markers. The UAV-based approach is demonstrated to be straightforward.
2. The UAV method provided data with unprecedented spatial resolution for the identification of recent small seismic offsets.
3. In the future, UAV-based high resolution data acquisition can be extensively applied to map active faults and assess seismic hazards.
